# Modifying a Paediatric Rational Prescribing Tool (POPI) for Use in the UK

**DOI:** 10.3390/healthcare7010033

**Published:** 2019-02-20

**Authors:** Fenella Corrick, Imti Choonara, Sharon Conroy, Helen Sammons

**Affiliations:** 1 Division of Medical Sciences & Graduate Entry Medicine, University of Nottingham, Royal Derby Hospital Centre, Uttoxeter Road, Derby DE22 3DT, UK; Sharon.Conroy@nottingham.ac.uk (S.C.); helen.sammons@nhs.net (H.S.); 2 North Devon District Hospital, Raleigh Park, Barnstaple EX31 4JB, UK

**Keywords:** paediatric, children, use of medicines, rational prescribing

## Abstract

Rational prescribing tools can be used by individual prescribers, organisations, and researchers to evaluate the quality of prescribing for research and quality improvement purposes. A literature search showed that there is only one tool for evaluating rational prescribing for paediatric patients in hospital and outpatient settings. The Pediatrics: Omission of Prescriptions and Inappropriate Prescriptions (POPI) tool was developed in France and comprises 105 criteria. The aim of this study was to modify this tool to facilitate its use in paediatric practice in the United Kingdom (UK). POPI criteria were compared to relevant UK clinical guidelines from the National Institute for Health and Care Excellence, the Scottish Intercollegiate Guideline Network and the British National Formulary for Children. Where guidelines differed, criteria were modified to reflect UK guidance. If there were no relevant guidelines or directly contradictory guidelines, criteria were removed. Overall, no change was made to 49 criteria. There were 29 modified to concord with UK guidelines. Four criteria were reduced to two criteria due to being linked in single guidelines. Twenty-three criteria were omitted, due to the absence of relevant UK guidance or directly conflicting UK practice, including one entire clinical category (mosquitos). One category title was amended to parallel UK terminology. The modified POPI (UK) tool comprises of eighty criteria and is the first rational prescribing tool for the evaluation of prescribing for children in hospital and outpatient settings in the UK.

## 1. Introduction

Rational prescribing describes practices aimed to optimise the use of medicines, encompassing safety, clinical effectiveness, access, and financial considerations. The WHO has defined rational prescribing as “when patients receive the appropriate medicines, in doses that meet their own individual requirements, for an adequate period of time, and at the lowest cost both to them and the community” [1]. Rational prescribing has been considered a problem mainly for low and lower middle-income countries, but it is increasingly being recognized as a problem in high-income countries [2,3].

Rational prescribing tools have been used, particularly in older adult medicine, as both research and quality improvement tools to investigate and improve rational prescribing [4]. These tools provide their users, whether individual prescribers, organizations, or research groups, with an objective measurement tool for the quality of prescribing according to rational prescribing principles. This facilitates research into factors involved in irrational prescribing, comparison across time or between groups of prescribers, organizations, services, or geographical regions, and the assessment of the efficacy of quality improvement interventions.

Children are a population particularly vulnerable to irrational prescribing due to the relative paucity of research supporting the paediatric use of medicines, with many medicines prescribed off-label, and children often excluded from drug trials.

The Pediatrics: Omission of Prescriptions and Inappropriate Prescriptions (POPI) tool was published in 2013 [5]. It was the first rational prescribing tool for use in paediatrics worldwide. The tool comprises explicit criteria based on French, American, and UK guidelines. The selection of clinical indications was based upon French prevalence data and the criteria were selected by Delphi consensus. In total, there are 105 “propositions” in the POPI tool, which are either indicators of potentially inappropriate prescriptions (for example, ineffective treatments) or potentially inappropriate omissions (such as highly effective first-line treatments).

Given the variation in the prevalence of disease, the availability of different formularies, and the diversity in paediatric practice internationally, the tool is not applicable outside of France. The only other extant rational prescribing tool for paediatric use is the potentially inappropriate prescribing in children (PIPc) indicators [6], which was developed exclusively for use in primary care settings. We therefore sought to modify the POPI tool for the application in UK paediatric practice in hospitals and outpatient settings by amending it to concord with UK clinical guidelines.

The aims of our study were twofold.

Firstly, to evaluate the applicability of the POPI tool to practice outside France by comparing the criteria to UK formulary and clinical guidelines.

Secondly, to modify the tool, where necessary, for application to UK paediatric practice and therefore to facilitate further evaluation of the tool using UK prescribing data. 

## 2. Materials and Methods

The 105 propositions of the POPI criteria were compared by one researcher (FC) to evidence-based UK clinical guidelines and clinical knowledge summaries from the National Institute of Health and Care Excellence (NICE) [7], the Scottish Intercollegiate Guidelines Network (SIGN) [8], the British National Formulary for Children (cBNF) [9], and the European Medicines Agency (EMA) [10]. The national guidance from NICE, SIGN and the cBNF were preferred; EMA recommendations were referred to when no national guidelines were available. This process used the most recent guidelines available on 1st October 2015. Where amendments were made, the specific related guideline is cited.

Following the comparison with the guidelines, there were three possible outcomes:

- Guidelines concurred with the POPI propositions. No change was made.
- There was partial discordance. POPI propositions were amended to match UK guidance.
- There was no guidance available or the proposition was in complete discordance with guidance, the proposition was omitted.

The final wording of the modified POPI criteria was reached as consensus in consultation with two paediatric clinical pharmacology consultants.

## 3. Results

Overall, no change was made to 49 propositions. There were 29 amended to concord more closely with UK guidelines. Four were reduced into two propositions, as they were closely related and the relevant guidelines referred to them together, simplifying the tool. Twenty-three were omitted altogether, which included the omission of an entire category. One category title was amended, as the diagnosis of attention deficit disorder without hyperactivity is not in use in the UK. 

The most substantial single change was the omission of the category of “mosquitos”. There are currently no areas in the UK where insect-borne diseases are endemic. This was not considered applicable to UK practice and therefore the category comprising of seven propositions, was removed. Some suggest that the viable habitat of mosquito vectors for vivax malaria may expand to the UK in the future, and if this were to occur, then this might be an appropriate area to target rational prescribing.

Twelve propositions were omitted due to a lack of relevant clinical guidelines (Table 1). The majority of these related to inappropriate prescriptions for medicines that are either not used in the UK, e.g., Diosmectite, or not used by the rectal route, e.g., rectal paracetamol.

Four propositions were also omitted where UK clinical guidelines contradicted the proposition. These are listed in Table 2 with the relevant conflicting UK guideline. They included the use of nitrofurantoin for urinary tract infections in young children; fluoride supplements in infants under the age of six months: the use of setrons (5-HT3 antagonists) for nausea/vomiting in association with chemotherapy; and isotretinoin for adolescent acne.

Two propositions were combined with closely related propositions, where the recommendations were linked in a single UK guideline in order to make the modified tool as concise as possible. The original and combined propositions are shown below in Table 3 with the related UK guidance. These related to the use of medicines for infants with bronchiolitis and the use of antibiotics in children with otitis media/upper respiratory tract infections.

There were 19 propositions that related to inappropriate prescriptions, and 10 propositions that related to inappropriate omissions that were amended to more closely concord with UK guidelines (Table 4 and Table 5). In some instances, the age was changed, e.g., loperamide is considered inappropriate in the UK in children under the age of four years old, whereas in France it is under the age of three years old. Some medicines such as benzyl benzoate are not recommended at all in children in the UK. Some medicines such as sodium cromoglycate are not recommended at all in France, whereas in the UK it can be used for exercise-induced asthma. In addition, the category title of “Attention deficit disorder with or without hyperactivity” was amended to “Attention deficit hyperactivity disorder”, as attention deficit disorder without hyperactivity is not recognised in UK clinical guidelines.

Others involved minor changes in relation to dosing and age for penicillin prophylaxis for children with sickle cell disease; patient groups for palivizumab: or vitamin use in infants.

The resulting modified POPI criteria therefore comprise 80 propositions assessing rational prescribing for children in accordance with up-to-date UK guidelines (see Table 6).

## 4. Discussion

The POPI criteria were modified to develop a list of potentially inappropriate prescriptions and omissions for children in the UK.

Over half of the propositions of the POPI criteria were altered. The majority of those changes were subtle modifications to bring the wording of propositions more closely in line with the specific wording of UK clinical guidelines. In other cases, the propositions were directly in contradiction of relevant guidelines and were amended accordingly. In order for this tool to be useful in appraising rational prescribing in the UK, it is important that prescribers are being measured against the specific standards they are striving for, and this would also facilitate straightforward interventions using UK guidelines for education and service improvement.

For 22 propositions, there were no relevant UK clinical guidelines. Absence from guidelines does not necessarily invalidate the recommendations of those propositions but the propositions were omitted, as they appeared to relate to the irrational use of medicines that do not appear to be prevalent in the UK. In some cases, the propositions related to medications not available in the UK. For instance, in the case of diosmectite for diarrhoea, there is some emerging evidence supporting its use [11] but this is not reflected in the availability of the product in the UK. 

In other cases, differing national practices may explain the absence if the type of irrational prescribing described is already rare in UK practise. This explanation likely underlies guidance about rectally administered drugs including paracetamol per rectum for pain and suppositories for cough. The cultural difference that may give rise to this variance in clinical practice was recognised in the European Medicines Agency Guideline on pharmaceutical development of medicines for paediatric use [12] when discussing medication acceptability in different countries, giving the example that “the rectal route of administration is not generally favoured in the UK”.

Two of the omitted propositions, in relation to sucrose for painful procedures in infants and nitrofurantoin as prophylaxis for urinary infection, may be absent from national UK guidelines because these are areas where there is not a national consensus of best practice. In reviewing these topics, local guidelines were found to differ, including some recommending nitrofurantoin for that purpose [13,14] and some preferring breast or bottle-feeding over sucrose, recommending contraindications and qualifying the guideline according to gestation and the age of the infant [15,16]. In the absence of a unifying national guideline on these topics, they were therefore not considered to be good candidates for screening prescribing practice nationally.

Four propositions were omitted due to the existence of UK clinical guidelines that were in direct conflict with the original proposition (see Table 2). 

Three of these appear to have been included as potentially inappropriate prescriptions in the original French tool due to the risk of interactions or side effects. One related to nitrofurantoin for the treatment of urinary infections. According to the report describing the development of the original POPI tool, this proposition was derived from a statement issued by AFSSAPS (the French Agency for the Safety of Health Products, Agence Française de Sécurité Sanitaire des Produits de Santé) in 2011, warning of cases of severe hepatic and pulmonary complications following long-term treatment with nitrofurantoin [17]. 

The cBNF does recommend monitoring liver function and for pulmonary symptoms if prescribing nitrofurantoin long-term, but it is licensed and indicated in acute uncomplicated urinary tract infections for children aged three months and older [9] and is second-line for children aged three months and older in the most recent NICE guideline NG109 [7]. 

The second related to isotretinoin and tetracycline antibiotics. This appears to be derived from a Good Practice Recommendation from AFSSAP describing isotretinoin as contraindicated with tetracyclines due to the reported occurrence of benign intracranial hyptertension with this combination [18]. This risk is recorded in the cBNF as a possible interaction, rated as “serious” with an anecdotal evidence base [9]. The combination is not recorded as a contraindication and combined topical retinoids and oral tetracyclines and recommended in the NICE Clinical Knowledge Summary. 

The third related to fluoride supplements before age six months. The related French guideline, an AFSAPPS statement in 2008, recommended that fluoride containing supplements such as toothpaste, commence when teeth erupt, on average at age six months [19]. This statement, like the relevant UK guidelines, discusses the risk of dental fluorosis with excess fluoride consumption during tooth development and recommends lower dose fluoride in toothpaste for young children. Both the NICE and SIGN guidelines quoted in Table 2 acknowledge the risk of dental fluorosis and state that the benefit of reduced caries favours starting fluoride supplementation as soon as teeth erupt with no definitive lower age limit of benefit to the child.

These all appear to reflect differing risk tolerance between the French and UK guidelines. In order that the modified tool reflects what is considered nationally to be good practice, the propositions were therefore omitted from the modified tool.

The fourth omitted proposition listed, “The use of setrons (5-HT3 antagonists) for chemotherapy-associated nausea and vomiting”, as a potentially inappropriate prescription. It was not clear what evidence was used to develop this proposition as none of the references in the report describing the development of the original tool related to chemotherapy-associated nausea and vomiting. One reference from the American Centers for Disease Control and Prevention recommended ondansetron as an anti-emetic for children [20]. It is possible that the inclusion of this criterion in the original tool constitutes a typographical error, and that it was intended to read as an inappropriate omission, given the importance of treating chemotherapy-associated nausea. It was therefore felt not to accurately reflect rational prescribing and was omitted from the modified tool.

Following the described amendments, the modified POPI(UK) tool comprises eighty criteria describing potentially inappropriate prescriptions or omissions. This tool is intended to evaluate the quality of prescribing for children in both hospital and outpatient settings, and is not limited to a specific group of prescribers. Similar tools for evaluating rational prescribing for older adults have facilitated a broad range of research, including research into quality of prescribing across different settings [21], studies into healthcare outcomes associated with irrational prescribing [22], and to predict adverse health outcomes in patient groups [23]. The tool is not intended for routine use by individual prescribers, as it requires experience to use.

## 5. Conclusions

The modified POPI (UK) criteria comprise the first screening tool available to assess rational prescribing for children in UK hospitals and outpatient settings. Clinical validation and reliability studies are needed and planned by the authors in order to evaluate the usability and reliability of this tool, which it is hoped will be used to study the rational use of medicines in children in the UK.

## Figures and Tables

**Table 1:** Propositions omitted due to the absence of relevant UK clinical guidelines.

Symptom or Illness Category	Omitted Paediatric Rational Prescribing Tool (POPI) Proposition
Pain and fever (inappropriate prescriptions).	Rectal administration of paracetamol as a first-line treatment.
Pain and fever (omission).	Failure to give sugar solution to newborn babies and infants under four months old two minutes prior to venipuncture.
Urinary infection (inappropriate prescription).	Nitrofurantoin used as a prophylactic.
Diarrhoea (inappropriate prescription).	The use of Diosmectite (Smecta) in combination with another medication [medication not approved for use in the UK].
	The use of Saccharomyces boulardii (Ultralevure) in powder form, or in a capsule that has to be opened prior to ingestion, to treat patients with a central venous catheter or an immunodeficiency.
	Intestinal antiseptics.
Cough (inappropriate prescription).	Mucolytic drugs, mucokinetic drugs, or helicidine before two years of age.
	Alimemazine (Theralene), oxomemazine (Toplexil), promethazine (Phenergan, and other types).
	Terpene-based suppositories.
Bronchiolitis (inappropriate prescription).	0.9% NaCl to relieve nasal congestion (not applicable if nasal congestion is already being treated with 3% NaCl delivered by a nebulizer).
ENT infections (inappropriate prescription).	Ethanolamine tenoate (Rhinotrophyl) and other nasal antiseptics.
Acne vulgaris (inappropriate prescription).	Androgenic progestins (levonorgestrel, norgestrel, norethisterone, lynestrenol, dienogest, contraceptive implants or vaginal rings).

**Table 2:** Propositions omitted due to conflicting UK clinical guidelines.

Symptom or Illness Category	Omitted POPI Proposition	Conflicting UK Guideline
Urinary infection (inappropriate prescription).	Nitrofurantoin used as a curative agent in children under six years of age, or indeed any other antibiotic if avoidable.	NICE guidance CG54: http://www.nice.org.uk/guidance/CG54/chapter/1-Guidance (Recommends nitrofurantoin for children aged three months and over.)
Vitamin supplements and antibiotic prophylaxis (inappropriate prescription)	Fluoride supplements prior to six months of age.	SIGN guidance 138: http://www.sign.ac.uk/pdf/SIGN138.pdf (Describes risks and benefits as balanced.) NICE Delivering Better Oral Health Toolkit: http://www.nice.org.uk/guidance/ph55/chapter/context#delivering-better-oral-health-toolkit (Recommends fluoride toothpaste as soon as teeth erupt.)
Nausea, vomiting, or gastroesophageal reflux (inappropriate prescription)	The use of setrons (5-HT3 antagonists) for chemotherapy-associated nausea and vomiting.	British National Formulary for Children: https://bnfc.nice.org.uk/drug/ondansetron.html (Chemotherapy-associated nausea and vomiting listed as licensed indication for ondansetron.)
Acne vulgaris (inappropriate prescription)	Isotretinoin in combination with a member of the tetracycline family of antibiotics.	NICE Acne Vulgaris Clinical Knowledge Summary: http://cks.nice.org.uk/acne-vulgaris#!topicsummary (Recommended second-line for moderate acne.)

**Table 3:** Propositions with shared UK guidelines and the simplified combined proposition.

Original POPI Propositions (Symptom or Illness Category)	Relevant UK Guidance (NICE, SIGN or cBNF)	Combined Proposition
Beta2 agonists, corticosteroids to treat an infant’s first case of bronchiolitis. (Bronchiolitis in infants, inappropriate prescription.)	NICE guidance NG9: http://www.nice.org.uk/guidance/ng9/chapter/1-Recommendations (Recommendation 1.4.3: Do not use any of the following to treat bronchiolitis in children: antibiotics; hypertonic saline; adrenaline (nebulised); salbutamol; montelukast; ipratropium bromide; systemic or inhaled corticosteroids; a combination of systemic corticosteroids and nebulised adrenaline.)	(Inappropriate prescription) Antibiotics, Beta2 agonists or corticosteroids to treat bronchiolitis.
Antibiotics in the absence of signs indicating a bacterial infection (acute otitis media, fever, etc.). (Bronchiolitis in infants, inappropriate prescription.)		
An antibiotic other than amoxicillin as a first-line treatment for acute otitis media, strep throat, or sinusitis (provided that the patient is not allergic to amoxicillin). An effective dose of amoxicillin for a pneumococcal infection is 80–90 mg/kg/day and an effective dose for a streptococcal infection is 50 mg/kg/day. (ENT infections, inappropriate prescription.)	NICE guidance CG69: http://www.nice.org.uk/guidance/cg69/chapter/1-Guidance (A no antibiotic prescribing strategy or a delayed antibiotic prescribing strategy should be agreed upon for patients with the following conditions: acute otitis media; acute sore throat/acute pharyngitis/acute tonsillitis; common cold; acute rhinosinusitis; acute cough/acute bronchitis. Depending on the clinical assessment of severity, patients in the following subgroups can also be considered for an immediate antibiotic prescribing strategy (in addition to a no antibiotic or a delayed antibiotic prescribing strategy): Bilateral acute otitis media in children younger than two years; acute otitis media in children with otorrhoea; acute sore throat/acute pharyngitis/acute tonsillitis when three or more Centor criteria are present. SIGN guideline 117: In severe cases, where the practitioner is concerned about the clinical condition of the patient, antibiotics should not be withheld. (Penicillin V 500 mg four times daily for 10 days is the dosage used in the majority of studies. A macrolide can be considered as an alternative first line treatment, in line with local guidance.)	(Inappropriate prescription) An antibiotic for <4 days symptoms of acute upper respiratory tract infection (except: bilateral acute otitis media in children younger than two years; acute otitis media in children with otorrhoea; acute sore throat/acute pharyngitis/acute tonsillitis when three or more Centor criteria are present).
Antibiotics for nasopharyngitis, congestive otitis, sore throat before three years of age, or laryngitis; antibiotics as a first-line treatment for acute otitis media showing few symptoms, before two years of age. (ENT infections, inappropriate prescription.)		

**Table 4:** Inappropriate prescription propositions modified to concord with UK guidelines.

Original POPI Propositions—Inappropriate Prescription (Symptom or Illness Category)	Relevant UK Guidance (NICE, SIGN or cBNF) (Recommendation)	Modified POPI Proposition—Inappropriate Prescription
Prescription of a medication other than paracetamol as a first-line treatment [for pain] (except in the case of migraine). (Pain and fever)	NICE Clinical Knowledge Summary: Management of mild-to-moderate pain: http://cks.nice.org.uk/analgesia-mild-to-moderate-pain#!scenario (Prescribe either paracetamol or ibuprofen alone. Both are suitable first-line choices for treating mild-to-moderate pain in children.)	Prescription of a medication other than paracetamol or ibuprofen as a first-line treatment for pain (except in the case of a migraine).
Oral solutions of ibuprofen administered in more than three doses per day using a graduated pipette of 10 mg/kg (other than Advil). (Pain and fever)	cBNF Ibuprofen: https://bnfc.nice.org.uk/drug/ibuprofen.html (Child 1–3 months 5 mg/kg 3–4 times daily. Child 3–6 months 50 mg 3 times daily; max. 30 mg/kg daily in 3–4 divided doses. Child 6 months to 1 year 50 mg 3–4 times daily; max. 30 mg/kg daily in 3–4 divided doses. Child 1–4 years 100 mg 3 times daily; max. 30 mg/kg daily in 3–4 divided doses. Child 4–7 years 150 mg 3 times daily; max. 30 mg/kg daily in 3–4 divided doses. Child 7–10 years old 200 mg 3 times daily; max. 30 mg/kg (max. 2.4 g) daily in 3–4 divided doses. Child 10–12 years 300 mg 3 times daily; max. 30 mg/kg (max. 2.4 g) daily in 3–4 divided doses. Child 12–18 years initially 300–400 mg 3–4 times daily; increased if necessary to max. 600 mg four times daily; maintenance dose of 200–400 mg three times daily may be adequate.)	Doses of ibuprofen administered in more than three doses per day or exceeding maximum dose of 30 mg/kg daily in three doses per day.
Gastric antisecretory drugs to treat gastroesophageal reflux, dyspepsia, the crying of newborn babies (in the absence of any other signs or symptoms), as well as faintness in infants. (Nausea, vomiting, or gastroesophageal reflux)	NICE guidance NG1: http://www.nice.org.uk/guidance/NG1/chapter/1-Recommendations (Recommendation 1.3.1: Do not offer acid-suppressing drugs, such as proton pump inhibitors (PPIs) or H_2_ receptor antagonists (H_2_RAs), to treat overt regurgitation in infants and children occurring as an isolated symptom. Recommendation 1.3.2: Consider a four-week trial of a PPI or H_2_RA for those who are unable to tell you about their symptoms (for example, infants and young children, and those with a neurodisability associated with expressive communication difficulties) who have overt regurgitation with one or more of the following: unexplained feeding difficulties (for example, refusing feeds, gagging or choking); distressed behaviour; faltering growth.)	Acid-suppressing drugs to treat overt regurgitation in the absence of feeding difficulties, distress, or faltering growth.
The use of type H2 antihistamines for long periods of treatment. (Nausea, vomiting, or gastroesophageal reflux)	NICE guidance NG1: http://www.nice.org.uk/guidance/NG1/chapter/1-Recommendations (Recommendation 1.3.4: four-week trial then stop, assess response, refer if symptoms recur.)	The use of H2 receptor antagonists for more than four weeks.
Erythromycin as a prokinetic agent. (Nausea, vomiting, or gastroesophageal reflux)	NICE guidance NG1: http://www.nice.org.uk/guidance/NG1/chapter/1-Recommendations (Do not offer metoclopramide, domperidone or erythromycin without seeking specialist advice.)	Erythromycin.
Loperamide before three years of age. (Diarrhoea)	cBNF Loperamide: https://bnfc.nice.org.uk/drug/loperamide-hydrochloride.html (Licensed from four years.)	Loperamide before four years of age
Antibiotic treatment for a sore throat, without a positive rapid diagnostic test result, in children less than three years old. (ENT infections)	SIGN guideline 117: http://www.sign.ac.uk/guidelines/fulltext/117/ (Minimises usefulness of rapid diagnostic test results in guiding therapy: In severe cases, where the practitioner is concerned about the clinical condition of the patient, antibiotics should not be withheld. (Penicillin V 500 mg four times daily for 10 days is the dosage used in the majority of studies. A macrolide can be considered as an alternative first-line treatment, in line with local guidance.)	Antibiotic treatment for a sore throat except in severe cases (where the patient’s clinical condition is documented as concerning).
Antibiotics to treat otitis media with effusion (OME), except in the case of hearing loss or if OME lasts for more than three months. (ENT infections)	NICE Clinical Knowledge Summary: http://cks.nice.org.uk/otitis-media-with-effusion#!scenario (Period of active observation for 6–12 weeks: During this period, do not prescribe antibiotics, steroids, antihistamines, decongestants, or mucolytics specifically for the treatment of otitis media with effusion (OME).)	Antibiotics to treat otitis media with effusion in the first 6–12 weeks.
H1-antagonists with sedative or atropine-like effects (pheniramine, chlorpheniramine), or camphor; inhalers, nasal sprays, or suppositories containing menthol (or any terpene derivatives) before 30 months of age. (ENT infections)	cBNF: https://www.evidence.nhs.uk/formulary/bnfc/current/3-respiratory-system/34-antihistamines-immunotherapy-and-allergic-emergencies/341-antihistamines#PHP11980 (Sedating antihistamines not for use in neonates, phenothiazine sedating antihistamines not for use <2 years, chlorphenamine not licensed <1 year.) https://www.evidence.nhs.uk/formulary/bnfc/current/3-respiratory-system/38-aromatic-inhalations (Menthol inhalations permissible, no sprays or suppositories in BNF nor terpene containing medicines.)	Sedating antihistamines (pheniramine, chlorpheniramine) before two years (except for anaphylaxis).
Ketotifen and other H1-antagonists, sodium cromoglycate. (Asthma)	SIGN guidance 141 (British guideline on the management of asthma): http://www.sign.ac.uk/pdf/SIGN141.pdf (Antihistamines and ketotifen are ineffective. Sodium cromoglycate for exercise-induced asthma.)	Ketotifen and other antihistamines.
The application of benzyl benzoate (Ascabiol) for periods longer than eight hours for infants and 12 h for children or for pregnant girls. (Scabies)	Children’s BNF: http://www.evidence.nhs.uk/formulary/bnf/current/13-skin/1310-anti-infective-skin-preparations/13104-parasiticidal-preparations/scabies and NICE Clinical Knowledge Summary: http://cks.nice.org.uk/scabies#!scenario (Benzyl benzoate should be avoided in children (permethrin or malathion are less irritant and more effective and should be used instead.)	Benzyl benzoate.
Treatment other than griseofulvin for Microsporum. (Ringworm)	NICE Clinical Knowledge Summary Fungal Skin infections: http://cks.nice.org.uk/fungal-skin-infection-body-and-groin#!scenario (Recommends topical treatment first-line. Gruseofulvin the only oral treatment appropriate for children.)	Oral treatment other than griseofulvin.
Any antibiotic other than mupirocin as a first-line treatment (except in cases of hypersensitivity to mupirocin). (Impetigo)	NICE Clinical Knowledge Summary Impetigo: http://cks.nice.org.uk/impetigo#!scenario (For localized [sic] infection, treat with topical fusidic acid… Topical mupirocin, retapamulin, and antiseptics are not recommended initially.)	Any antibiotic other than fusidic acid as a first-line treatment (except in cases of hypersensitivity to fusidic acid).
Orally administered acyclovir to treat primary herpetic gingivostomatitis. (Herpes simplex)	NICE Clinical Knowledge Summary Herpes Simplex (oral): http://cks.nice.org.uk/herpes-simplex-oral#!scenario:1 (Consider oral antivirals for immunocompetent individuals with severe gingivostomatitis.)	Orally administered aciclovir to treat severe herpetic gingivostomatitis.
A strong dermocorticoid (clobetasol propionate 0.05% Dermoval, betamethasone dipropionate Diprosone) applied to the face, the armpits or groin, and the backside of babies or young children. (Atopic eczema)	NICE guidance CG57: https://www.nice.org.uk/guidance/CG57/chapter/1-Guidance (use mild potency for the face and neck, except for short-term (3–5 days) use of moderate potency for severe flares; use moderate or potent preparations for short periods only (7–14 days) for flares in vulnerable sites such as axillae and groin; do not use very potent preparations in children without specialist dermatological advice.) Children’s BNF Dermoval not listed: http://www.evidence.nhs.uk/formulary/bnf/current/13-skin/134-topical-corticosteroids/topical-corticosteroid-preparation-potencies	A potent topical corticosteroid applied to the face, or for >14 days applied to the axilla or groin.
Local or systemic antihistamine during the treatment of outbreaks. (Atopic eczema)	NICE guidance CG57: https://www.nice.org.uk/guidance/CG57/chapter/1-Guidance (Recommendation 1.5.6: Healthcare professionals should offer a 1-month trial of a non-sedating antihistamine to children with severe atopic eczema or children with mild or moderate atopic eczema where there is severe itching or urticaria. Healthcare professionals should offer a 7–14 day trial of an age-appropriate sedating antihistamine to children aged 6 months or over during an acute flare of atopic eczema if sleep disturbance has a significant impact on the child or parents or carers.)	Prescription of antihistamines except as a trial for severe itching or where sleep disturbance has a significant impact on the child or carers.
Cyproheptadine (Perlactin), clonidine. (Anorexia)	NICE guidance CG9: https://www.nice.org.uk/guidance/CG9/chapter/1-Guidance (Recommendation 1.2.3.1: Medication should not be used as the sole or primary treatment for anorexia nervosa.)	Prescription of medications as a sole or primary treatment for anorexia nervosa.
Antipsychotic drugs to treat attention deficit disorder without hyperactivity. (attention deficit disorder with or without hyperactivity)	NICE guidance CF72: https://www.nice.org.uk/guidance/cg72/chapter/1-Guidance (Recommendation 1.5.5.7: Antipsychotics are not recommended for the treatment of ADHD in children and young people.)	Antipsychotic drugs to treat attention deficit hyperactivity disorder.
Slow release methylphenidate as two doses per day, rather than only one dose. (Attention deficit disorder with or without hyperactivity)	NICE guidance CF72: https://www.nice.org.uk/guidance/cg72/chapter/1-Guidance (Recommendation 1.8.2.2: modified-release preparations should be given as a single dose in the morning.)	Modified release methylphenidate as two doses per day rather than only one dose.

**Table 5:** Omission of prescription propositions modified to concord with UK guidelines.

Original POPI Propositions—Inappropriate Omission (Symptom or Illness Category)	Relevant UK Guidance (NICE, SIGN or cBNF) (Recommendation)	Modified POPI Proposition—Inappropriate Omission
Insufficient intake of vitamin D. Minimum vitamin D intake: Breastfed baby = 1000 to 1200 IU/day; Infant, 18 months of age (milk enriched in vitamin D) = 600–800 IU/day; Child aged between 18 months and five years, and adolescents aged between 10 and 18 years: two quarterly loading doses of 80,000 to 100,000 IU/day in winter (adolescents can take this dose in one go). (Vitamin supplements and antibiotic prophylaxis)	NICE guidance PH56: http://www.nice.org.uk/guidance/ph56/chapter/1-Recommendations (Vitamin D supplements should be available for at-risk groups, including infants and children <5 years, Healthy Start vitamins.)	Healthy Start vitamins for infants and children 0.5–5 years or having less than 500 mL infant formula per day.
Antibiotic prophylaxis with phenoxymethylpenicillin (Oracilline) starting from two months of age and lasting until five years of age for children with sickle-cell anaemia: 100,000 IU/kg/day (in two doses) for children weighing 10kg or less and 50,000 IU/kg/day for children weighing over 10 kg (also in two doses). (Vitamin supplements and antibiotic prophylaxis)	NICE Clinical Knowledge Summary: http://cks.nice.org.uk/sickle-cell-disease#!scenario:3 (Explain that lifelong prophylaxis is recommended, but it is particularly important that there is full adherence up to five years of age. Prescribe phenoxymethylpenicillin (penicillin V) prophylaxis from the age of one month, at a dose of: 125 mg twice a day for infants and children up to five years of age. 250 mg twice a day for children from six to 12 years of age. 500 mg twice a day for adults and children older than 12 years of age. Erythromycin is recommended for people who are allergic to penicillin, at a dose of: 125 mg twice a day for infants and children up to two years of age. 250 mg twice a day for adults and children older than two years of age.)	Antibiotic prophylaxis with phenoxyethylpenicillin (penicillin V) from age one month until five years old for children with sickle-cell anaemia at a dose of: 125 mg twice a day for infants and children up to five years of age. 250 mg twice a day for children from six to 12 years of age. 500 mg twice a day for adults and children older than 12 years of age. Or Erythromycin for children who are allergic to penicillin, at a dose of: 125 mg twice a day for infants and children up to two years of age. 250 mg twice a day for children older than two years of age.
Oral rehydration solution. (Nausea, vomiting, or gastroesophageal reflux)	NICE guidance CG84: http://www.nice.org.uk/guidance/cg84/chapter/1-Guidance#fluid-management (Offer ORS solution as supplemental fluid to children at risk of dehydration or use in dehydrated children unless IV fluid is indicated.)	Amend: Oral rehydration solution for dehydrated children unless IV fluid therapy is indicated (shock, red flag symptoms despite ORS, persist vomiting of ORS).
Oral rehydration solution. (Diarrhoea)	NICE guidance CG84: http://www.nice.org.uk/guidance/cg84/chapter/1-Guidance#fluid-management (Offer ORS solution as supplemental fluid to children at risk of dehydration or use in dehydrated children unless IV fluid is indicated.)	Amend: Oral rehydration solution for dehydrated children unless IV fluid therapy is indicated (shock, red flag symptoms despite ORS, persistent vomiting of ORS).
Failure to propose a whooping cough booster vaccine for adults who are likely to become parents in the coming months or years (only applicable if the previous vaccination was more than 10 years ago). This booster vaccination should also be proposed to the family and entourage of expectant parents (parents, grandparents, nannies/child minders). (Cough).	NICE CKS Antenatal care of uncomplicated pregnancy: http://cks.nice.org.uk/antenatal-care-uncomplicated-pregnancy#!scenario (28 weeks gestation: Offer vaccination against pertussis.)	Amend: Failure to propose a whooping cough vaccine for pregnant women.
Palivizumab in the following cases: (1) babies born both at less than 35 weeks of gestation and less than six months prior to the onset of a seasonal RSV epidemic; (2) children less than two years old who have received treatment for bronchopulmonary dysplasia in the past six months; (3) children less than two years old suffering from congenital heart disease with hemodynamic abnormalities. (Bronchiolitis in infants).	SIGN guidance 91 (Bronchiolitis in children): http://www.sign.ac.uk/guidelines/fulltext/91/index.html (… recommends use of palivizumab in high risk groups, as defined by the committee (children under two years of age with chronic lung disease, on home oxygen or who have had prolonged use of oxygen; infants less than six months of age who have left to right shunt haemodynamically significant congenital heart disease and/or pulmonary hypertension; children under two years of age with severe congenital immuno-deficiency).)	Amend: Palivizumab in high-risk cases, defined as: children <2 years with chronic lung disease on home oxygen or who have prolonged use of oxygen; infants <6 months with left-to-right shunt haemodynamically significant congenital heart disease and/or pulmonary hypertension; children <2 years with severe congenital immunodeficiency.)
Asthma inhaler appropriate for the child’s age. (Asthma)	NICE guidance TA10: https://www.nice.org.uk/guidance/ta10 (NICE has recommended that for children under the age of five years who have chronic stable asthma: both corticosteroids and bronchodilator therapy should routinely be delivered by Pressurised Metered Dose Inhaler (pMDI) and spacer system, with a facemask where necessary. Where this combination is not clinically effective for the child, and depending on the child’s condition, nebulised therapy may be considered and in the case of children aged 3–5 years, a dry powder inhaler (DPI) may also be considered. The choice of which pMDI device and spacer to use should be determined by the specific needs of the child and how well it works for them. Once these factors have been taken into account the choice should be made on the basis of reducing costs.)	Amend: Asthma inhaler appropriate for the child’s age (aged <5 years, either Metered Dose Inhaler with spacer system or nebuliser; age 3–5 years Dry Powder Inhaler may be appropriate).
Contraception (provided with a logbook/diary) for menstruating girls taking isotretinoin. (Acne vulgaris)	Children’s BNF: https://www.evidence.nhs.uk/formulary/bnf/current/13-skin/136-acne-and-rosacea/1362-oral-preparations-for-acne/oral-retinoid-for-acne/isotretinoin (Effective contraception must be used.)	Amend: Contraception for menstruating girls taking isotretinoin.
A second dose of ivermectin two weeks after the first. (Scabies)	Children’s BNF: https://bnfc.nice.org.uk/treatment-summary/skin-infections.html (Ivermectin only available by special order, unlicensed for scabies.) https://bnfc.nice.org.uk/drug/permethrin.html https://bnfc.nice.org.uk/drug/malathion.html (Apply once weekly for two doses.)	Amend: A second application of permethrin or malathion one week after the first.
Decontamination of household linen and clothes and treatment for other family members. (Scabies)	NICE Clinical Knowledge Summary: http://cks.nice.org.uk/scabies#!scenario (Decontamination of household linen and clothes and same day treatment of all members of the household.)	Amend: Decontamination of household linen and clothes and same day treatment of all members of the household.

**Table 6:** The modified POPI (UK) tool.

**DIVERSE ILLNESSES**
PAIN AND FEVER
Inappropriate prescriptions.
Prescription of two alternating antipyretics as a first-line treatment.
Prescription of a medication other than paracetamol or ibuprofen as a first-line treatment for pain (except in the case of a migraine).
The combined use of two NSAIDs.
Doses of ibuprofen administered in more than three doses per day or exceeding maximum dose of 30 mg/kg daily in three doses per day.
Opiates to treat migraine attacks.
Omissions.
Failure to give an osmotic laxative to patients being treated with morphine for a period of more than 48 h.
URINARY INFECTIONS
Inappropriate prescriptions.
Antibiotic prophylaxis following an initial infection without complications (except in the case of uropathy).
Antibiotic prophylaxis in the case of asymptomatic bacterial infection (except in the case of uropathy).
VITAMIN SUPPLEMENTS AND ANTIBIOTIC PROPHYLAXIS
Omissions.
Healthy Start vitamins for infants and children 0.5–5 years or having less than 500 mL infant formula per day.
Antibiotic prophylaxis with phenoxyethylpenicillin (penicillin V) from age one month until five years for children with sickle-cell anaemia at a dose of: 125 mg twice a day for infants and children up to five years of age. 250 mg twice a day for children from six to 12 years of age. 500 mg twice a day for adults and children older than 12 years of age. Or Erythromycin for children who are allergic to penicillin, at a dose of: 125 mg twice a day for infants and children up to two years of age. 250 mg twice a day for children older than two years of age.
**DIGESTIVE PROBLEMS**
NAUSEA, VOMITING, OR GASTROESOPHAGEAL REFLUX
Inappropriate prescriptions.
Metoclopramide.
Domperidone.
Oral administration of an intravenous proton pump inhibitor (notably by nasogastric tube).
Acid-suppressing drugs to treat overt regurgitation in the absence of feeding difficulties, distress, or faltering growth.
The combined use of proton pump inhibitors and NSAIDs, for a short period of time, in patients without risk factors.
The use of H2 receptor antagonists for more than four weeks.
Erythromycin.
Omissions.
Oral rehydration solution (ORS) for dehydrated children unless IV fluid therapy is indicated (shock, red flag symptoms despite ORS, persist vomiting of ORS).
DIARRHOEA
Inappropriate prescriptions.
Loperamide before four years of age.
Loperamide in the case of invasive diarrhoea.
Omissions.
Oral rehydration solution (ORS) for dehydrated children unless IV fluid therapy is indicated (shock, red flag symptoms despite ORS, persist vomiting of ORS).
**ENT-PULMONARY PROBLEMS**
COUGH
Inappropriate prescriptions.
Pholcodine.
Omissions.
Failure to propose a whooping cough vaccine for pregnant women.
BRONCHIOLITIS IN INFANTS
Inappropriate prescriptions.
Antibiotics, Beta2 agonists or corticosteroids to treat bronchiolitis.
H1-antagonists, cough suppressants, mucolytic drugs, or ribavirin to treat bronchiolitis.
Omissions.
Palivizumab in high-risk cases, defined as: Children <2 years with chronic lung disease on home oxygen or who have prolonged use of oxygen; Infants <6 months with left-to-right shunt haemodynamically significant congenital heart disease and/or pulmonary hypertension; Children <2 years with severe congenital immunodeficiency.
ENT INFECTIONS
Inappropriate prescriptions.
An antibiotic for <4 days symptoms of acute upper respiratory tract infection (except: Bilateral acute otitis media in children younger than two years; Acute otitis media in children with otorrhoea; Acute sore throat/acute pharyngitis/acute tonsillitis when three or more Centor criteria are present.)
Antibiotic treatment for a sore throat except in severe cases (anticipated to be no more than 20% of cases).
Antibiotics to treat otitis media with effusion in the first 6–12 weeks.
Corticosteroids to treat acute suppurative otitis media, nasopharyngitis, or strep throat.
Nasal or oral decongestant (oxymetazoline (Aturgyl), pseudoephedrine (Sudafed), naphazoline (Derinox), ephedrine (Rhinamide), tuaminoheptane (Rhinofluimicil), phenylephrine (Humoxal)).
Sedating antihistamines (pheniramine, chlorpheniramine) before two years (except for anaphylaxis).
Eardrops in the case of acute otitis media.
Omissions.
Doses in mg for drinkable (solutions of) amoxicillin or josamycin.
Paracetamol combined with antibiotic treatment for ear infections to relieve pain.
ASTHMA
Inappropriate prescriptions.
Ketotifen and other antihistamines.
Cough suppressants.
Omissions.
Asthma inhaler appropriate for the child’s age (aged <5 years, either Metered Dose Inhaler with spacer system or nebuliser; age 3–5 years Dry Powder Inhaler may be appropriate).
Preventative treatment (inhaled corticosteroids) in the case of persistent asthma.
**DERMATOLOGICAL PROBLEMS**
ACNE VULGARIS
Inappropriate prescriptions.
Minocycline.
The combined use of an oral and a local antibiotic.
Oral or local antibiotics as a monotherapy (not in combination with another drug).
Cyproterone + ethinylestradiol (Diane 35) as a contraceptive to allow isotretinoin per os.
Omissions.
Contraception for menstruating girls taking isotretinoin.
Topical treatment (benzoyl peroxide, retinoids, or both) in combination with antibiotic therapy.
SCABIES
Inappropriate prescriptions.
Benzyl benzoate.
Omissions.
A second application of permethrin or malathion one week after the first.
Decontamination of household linen and clothes and same day treatment of all members of the household.
LICE
Inappropriate prescriptions.
The use of aerosols for infants, children with asthma, or children showing asthma-like symptoms such as dyspnea.
RINGWORM
Inappropriate prescriptions.
Oral treatment other than griseofulvin.
Omissions.
Topical treatment combined with an orally administered treatment.
Griseofulvin taken during a meal containing a moderate amount of fat.
IMPETIGO
Inappropriate prescriptions.
The combination of a locally applied and orally administered antibiotic.
Fewer than two applications per day for topical antibiotics.
Any antibiotic other than fusidic acid as a first-line treatment (except in cases of hypersensitivity to fusidic acid).
HERPES SIMPLEX
Inappropriate prescriptions.
Topical agents containing corticosteroids.
Topical agents containing aciclovir before six years of age.
Omissions.
Paracetamol during an outbreak of herpes.
Orally administered aciclovir to treat severe herpetic gingivostomatitis.
ATOPIC ECZEMA
Inappropriate prescriptions.
A potent topical corticosteroid applied to the face, or for >14 days applied to the axilla or groin.
More than one application per day of a dermocorticoid, except in cases of severe lichenification.
Prescription of antihistamines except as a trial for severe itching or where sleep disturbance has a significant impact on the child or carers.
Topically applied 0.03% tacrolimus before two years of age.
Topically applied 0.1% tacrolimus before 16 years of age.
Oral corticosteroids to treat outbreaks.
**NEUROPSYCHIATRIC DISORDERS**
EPILEPSY
Inappropriate prescriptions.
Carbamazepine, gabapentin, oxcarbazepine, phenytoin, pregabalin, tiagabine, or vigabatrin in the case of myoclonic epilepsy.
Carbamazepine, gabapentin, oxcarbazepine, phenytoin, pregabalin, tiagabine, or vigabatrin in the case of epilepsy with absence seizures (especially for childhood absence epilepsy or juvenile absence epilepsy).
Levetiracetam, oxcarbamazepine in mL or in mg without systematically writing XX mg per Y mL.
DEPRESSION
Inappropriate prescriptions.
An SSRI antidepressant other than fluoxetine as a first-line treatment (in the case of pharmacotherapy).
Tricyclic antidepressants to treat depression.
NOCTURNAL ENURESIS
Inappropriate prescriptions.
Desmopressin administered by a nasal spray.
Desmopressin in the case of d`aytime symptoms.
An anticholinergic agent used as a monotherapy in the absence of daytime symptoms.
Tricyclic agents in combination with anticholinergic agents.
Tricyclic agents as a first-line treatment.
ANOREXIA
Inappropriate prescriptions.
Prescription of medications as a sole or primary treatment for anorexia nervosa.
ATTENTION DEFICIT DISORDER WITH HYPERACTIVITY
Inappropriate prescriptions.
Pharmacological treatment before age six (before school), except in severe cases.
Antipsychotic drugs to treat attention deficit hyperactivity disorder.
Modified release methylphenidate as two doses per day, rather than only one dose.
Omissions.
Recording a growth chart (height and weight) if the patient is taking methylphenidate.
